# Development of a novel cognitive-motor integration balance assessment in healthy young adults: a pilot study

**DOI:** 10.3389/fnint.2025.1680294

**Published:** 2026-01-14

**Authors:** Sara E. Weinberg, Nicole Smeha, Lauren E. Sergio, Taylor W. Cleworth

**Affiliations:** 1 School of Kinesiology and Health Science, York University, Toronto, ON, Canada; 2 Michael G. DeGroote School of Medicine, Faculty of Health Sciences, McMaster University, Hamilton, ON, Canada

**Keywords:** balance, cognitive-motor integration, motor control, postural control, sensory, vestibular, whole-body

## Abstract

Many skills necessary to perform activities of daily living require individuals to think and move at the same time; otherwise known as cognitive-motor integration (CMI). An upper extremity CMI task has shown how CMI performance changes with age, neurotrauma, and sport experience; however, the majority of movements required for activities of daily living extend beyond the upper extremity. Therefore, the purpose of this pilot study was to compare a full-body balance-related CMI task with the validated upper extremity task. Twenty-nine young healthy adults [24.3 ± 5.1 years (SD); 12 females] completed 2 CMI tasks to assess upper extremity CMI and full-body CMI. In general, both CMI tasks varied in difficulty, ranging from congruent interactions with targets, to incongruent interactions which included visual feedback reversal (requiring increased CMI). Performance in both tasks were quantified using reaction time (RT), movement time (MT), and normalized path length (nPL). An interaction effect of task and condition was found for MT [*F*_(1_,_28)_ = 9.344, *p* = 0.005] and nPL [*F*_(1_,_28)_ = 12.766, *p* = 0.001], with larger increases across conditions in the full-body task compared to the upper extremity task. For the upper extremity task, sex predicted RT, where males had quicker RTs than females (unstandardized *B* = –78.968, *p* = 0.038). For the full-body task, MT and nPL were predicted by age and sport experience, respectively; where younger age resulted in faster MTs (unstandardized *B* = 235.546, *p* = 0.009), and more sport experience led to less variable nPLs (unstandardized *B* = –3.802, *p* = 0.005). Lastly, the full-body task found that sport experience was moderated by sex (unstandardized *B* = 203.650, *p* = 0.014), where only females saw decreases in MT with increasing sport experience. The full-body CMI task provides a more comprehensive analysis of sensory, motor, and cognitive contributions to coordination tasks. An isolated upper extremity task may be limited in its ability to extract meaningful information that could contribute to difficulties in performing activities of daily living. Future work could utilize this task in clinical populations with the potential to uncover differences that might not be apparent in standard assessment protocols.

## 1 Introduction

Many skills that are necessary to perform activities of daily living require individuals to think and move at the same time; otherwise known as cognitive-motor integration (CMI). Previous work has shown that deficits are not always apparent when performing motor or cognitive tasks separately (Huang and Mercer, 2001). Thus, an upper extremity CMI assessment task has been developed and validated to demonstrate how CMI performance changes with age, neurotrauma, and sport experience. Prior research has shown that CMI performance is worse in older individuals compared to younger individuals (Echlin et al., 2020). In addition, when comparing those with concussion to controls, CMI performance is poorer when assessing timing and accuracy measures (Sergio et al., 2020). Subsequent work has further demonstrated that individuals with concussion history and higher sport experience outperform those with concussion history and less sport experience (Dalecki et al., 2019). It has therefore been suggested that sport experience might provide brain network resilience that can compensate for concussion-related declines in CMI performance. Additionally, previous eye-hand coordination tasks have reported sex-related differences. In difficult conditions, when an individual is forced to prioritize speed or accuracy to complete the task, movement biases emerge, whereby males favor speed and females favor accuracy (Rohr, 2006). Finally, CMI performance has also been shown to differ between males and females, where males were less accurate when performing the task (Rogojin et al., 2019).

While upper extremity CMI tasks demonstrate changes associated with age, concussion, and sport experience, the majority of movements required to perform daily activities extend beyond the upper extremity. For example, to retrieve an item from a grocery shelf, you must be able to reach and grasp, as well as maintain your balance. In order to accurately perform this goal-directed movement, the motor system must be provided with sufficient sensory information. While an isolated upper extremity task relies heavily on input from the visual and proprioceptive systems (Sabes, 2011), a full-body task involves whole-body movements which have vestibular input playing a more prominent role. The vestibular system consists of a sensory organ in your inner ear that detects linear and angular acceleration to help maintain balance. It works in conjunction with the visual and proprioception systems to provide essential information about head and body position, as well as self-motion (Valko et al., 2012). Therefore, incorporating the CMI paradigm into a task that involves whole-body movements has the potential to reveal deficits not always detected through other assessments.

Research assessing how cognitive tasks influence balance has shown that the typical dual-task approach has its limitations. Dual-task paradigms often assess an individual’s balance when they are asked to perform a secondary cognitive task (Saberi et al., 2024). However, this may lead to task prioritization, where either the balance task or cognitive task is prioritized. The CMI approach used in this study requires the cognitive component to be integrated with the motor component, eliminating the ability for individuals to prioritize one task over the other. The addition of a full-body CMI assessment would provide a more comprehensive analysis while remaining an ecologically valid tool to assess CMI. Therefore, the purpose of this pilot study is two-fold; firstly, it will examine how movements requiring CMI impact performance, secondly, it will compare the full-body CMI balance task with the validated upper extremity task. It was hypothesized that there will be worse performance (slower reaction times, longer movement times, and more variable paths) in the conditions requiring increased CMI, as well as decreased performance in the full-body task compared to the upper extremity task, independent of condition. Furthermore, it was suggested that younger individuals, males, and those with more sport experience would outperform their counterparts.

## 2 Materials and methods

### 2.1 Participants

Twenty-nine young healthy adults [24.3 ± 5.1 years (SD); 12 females] were recruited to participate in the study (Table 1). Inclusion criteria consisted of individuals between the ages of 18–40 years old. Exclusion criteria included any self-reported neurological or orthopedic impairments which could affect their ability to perform the tasks. Study protocol was approved in accordance with York University’s Ethics Review Board and participants provided informed consent prior to participating in the study.

**TABLE 1:** Summary of participant demographics (*n* = 29).

Variables	*N*	Mean (SD)
Age (years)		24.3 (5.1)
Sex		
Female	12	
Male	17	
Height (inches)		67.9 (3.5)
Weight (lbs)		158.1 (28.4)
Ethnicity		
White	12	
South Asian	3	
East Asian	2	
Southeast Asian	1	
Middle Eastern	3	
West Indian	2	
Latin American	1	
Multiracial	4	
Not reported	1	
Concussion history		
Yes	7	
No	22	
Sport experience (years)		15.6 (8.4)
Soccer	10	
Basketball	8	
Volleyball	8	
Tennis/pickleball	6	
Hockey	6	
Swimming	5	
Combat sports	5	
Running/cycling	4	
Other (*n* < 4 per group)	24	
Highest level of education		
High school degree	12	
Bachelor’s degree	7	
Master’s degree	10	
Occupation		
Student	27	
Full-time employee	2	

SD = standard deviation.

### 2.2 Procedure

#### 2.2.1 Questionnaire

Demographic information was collected on participants’ age, sex, sport experience, years of education, occupation, and touchscreen/video game use. Furthermore, health-related questionnaires were used to collect information on concussion history, medication use, and family history of neurological disorders.

#### 2.2.2 Behavioral tasks

**FIGURE 1:**
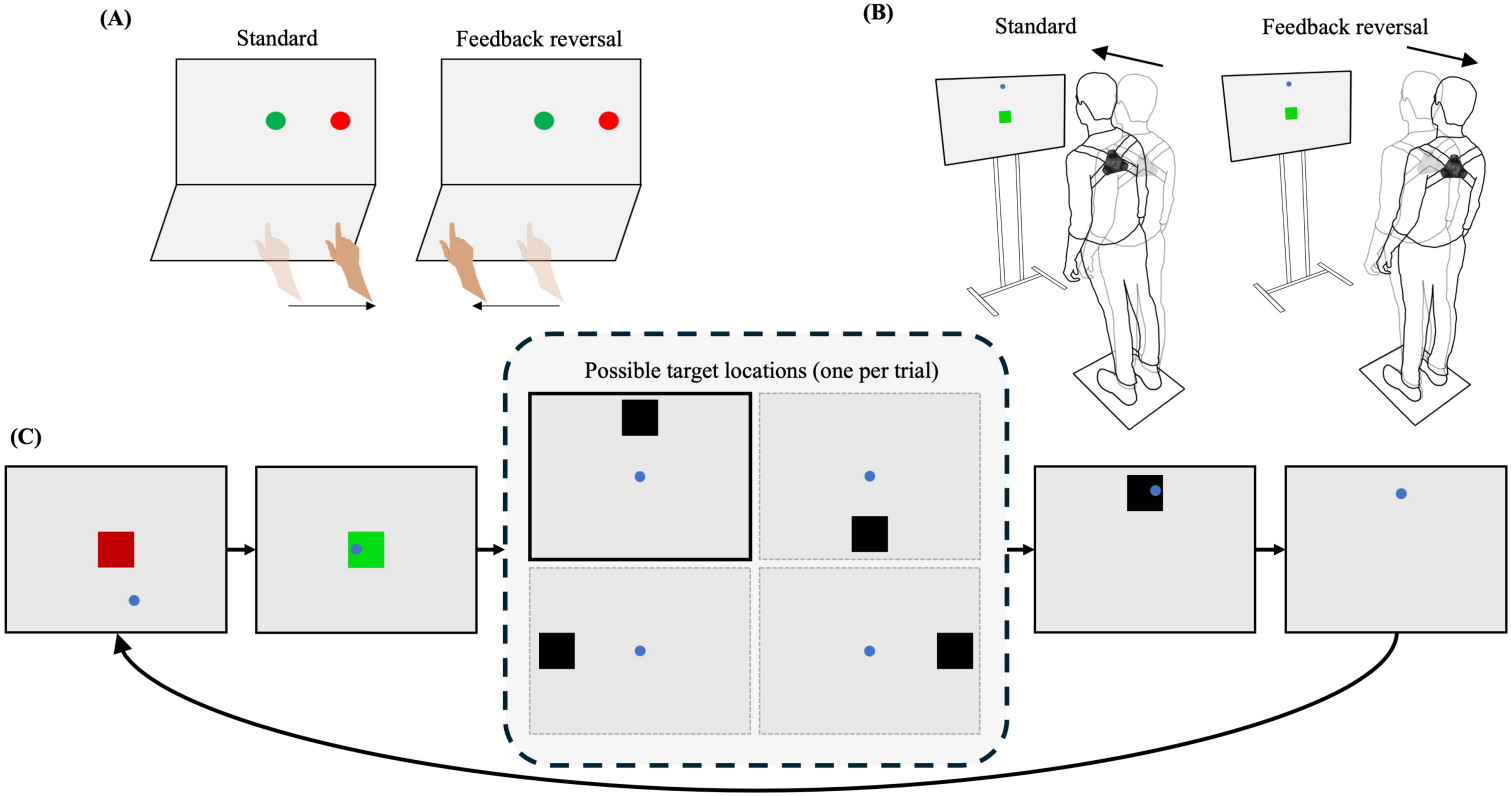
Experimental setup for **(A)** upper extremity task and **(B)** full-body task. **(C)** Order of events for each trial in the full-body CMI task (similar protocol used in the upper extremity CMI task): Participants moved the cursor to the central target, which would turn green when the cursor was on target, one of the four peripheral targets appeared and the central target disappeared, participants moved the cursor onto the peripheral target, the target would disappear and the trial ended.

Two CMI assessment tasks were implemented in this study. A laptop and touchscreen were used to assess upper extremity CMI, and a television monitor and 3D motion capture were used to assess full-body CMI. In both tasks, the goal was to move the cursor from a central target to one of four peripheral targets (up, down, left, and right) as quickly and accurately as possible. In general, both CMI tasks varied in difficulty, ranging from congruent interactions with targets, to incongruent interactions which included visual feedback reversal (requiring increased CMI). The two CMI tasks were counterbalanced across participants with adequate rest periods provided to prevent fatigue. Within each task, conditions were presented in a randomized order and each condition consisted of 20 trials total, with 5 pseudo-randomly presented trials to each of the 4 peripheral targets. Practice trials (one to each target) for each condition were included in order to ensure task comprehension. The order of events for each trial were as follows: (1) A central target was presented on screen, (2) participants moved the cursor to the central target, which would turn green when the cursor was on target, (3) once 4,000 ms had elapsed with the cursor on target, one of the four peripheral targets appeared and the central target disappeared, (4) participants were instructed to move the cursor toward the peripheral target, (5) once the cursor was on the peripheral target and 500 ms had elapsed, the target would disappear and the trial ended, and (6) An inter-trial interval of 2,000 ms would occur before the central target would reappear (Figure 1).

##### 2.2.2.1 Upper extremity CMI task

In the upper extremity CMI task, the participant would interact with an external touchscreen oriented orthogonal to the laptop screen. This task consisted of two conditions: (1) Standard, with finger movements in the same direction as visual information and (2) Feedback reversal, where the visual feedback was rotated 180° and finger movements were in the opposite direction of visual information, requiring increasing CMI (Figure 1). The peripheral targets were 15 mm in diameter and were placed 55 mm from the central target.

##### 2.2.2.2 Full-body CMI task

In the full-body CMI task, participants stood on a force plate facing a television monitor (1.10 × 0.62 m) located at eye level, approximately 1.27 m away. A 3-dimensional motion capture tracker (Vive Tracker, HTC Corp., Taiwan) was attached to the trunk of the participant and was used to update the position of the cursor on the monitor (Figure 1). While controlling the cursor, the participants were instructed to stand with their feet in place (foot width standardized to foot length) and to lean at the ankle joint, without bending at the knees or hip. This task consisted of two conditions: (1) Standard, where a forward lean corresponded to the cursor moving up on the screen, a backward lean would drive the cursor downward, and a leftward and rightward lean would move the cursor to the left and right, respectively; and (2) Feedback reversal, where the visual feedback was rotated 180° (ex. forward lean would move the cursor downwards), requiring increasing CMI.

The peripheral targets were 20 × 20 mm, and target locations were individualized for each participant. Calibration trials were utilized to find the participants’ maximum displacement in each of the four directions (forward, backward, leftward, and rightward) and the targets were placed at 80% of these values. This individualized approach ensured that task difficulty was consistent across participants.

### 2.3 Measurements

Ground reaction forces and moments were recorded from the force plate (AMTI, United States) at 100 Hz (Power 1401, Spike2, CED, United Kingdom) and used to calculate anterior-posterior and mediolateral center of pressure displacements. Kinematic measures of the cursor’s x and y coordinates were collected for both tasks. Cursor position was sampled at 50 Hz (upper extremity task) and 100 Hz (full-body task), and low pass filtered with a 10 Hz (upper extremity task) and 5 Hz (full-body task) dual-pass Butterworth filter. For each trial, movement onset was calculated as the first timepoint that the cursor (upper extremity task) or center of pressure (full-body task) trajectory exceeded 10% peak velocity and ballistic movement offset (upper extremity task) was the timepoint at which the trajectory went below 10% peak velocity. Total movement offsets occurred when the cursor entered the target for the final time (full-body task) or the final cross of 10% peak velocity once the cursor was within the target boundaries (upper extremity task).

### 2.4 Outcome measures

Performance in both tasks were quantified by calculating reaction time (RT), movement time (MT), and normalized path length (nPL) for each trial. RT was the time interval between trial onset (appearance of a peripheral target) and movement onset (when movement crossed 10% of peak velocity). MT was the time interval between movement onset and the end of the movement (when the cursor entered the peripheral target for at least 500 ms). nPL was the percentage of distance covered between movement onset and the end of the movement, relative to a straight line between starting and endpoints.

### 2.5 Statistical analysis

2 × 2 repeated-measures analysis of variance (ANOVAs) were conducted to examine the effect of task (upper extremity vs. full-body) and condition (standard vs. feedback reversal) for RT, MT, and nPL. Multiple linear regressions were performed to determine the association between exposure variables (age and sport experience) and outcome variables (RT, MT, and nPL) for the upper extremity and full-body tasks, and whether the relationship is moderated by sex or concussion history. Any models that included a significant moderator variable were then assessed using a simple slopes analysis. All statistical tests were performed using SPSS (IBM Corp., N.Y., United States).

Shapiro-Wilks tests were used to assess normality. While data transformations (log, square-root, and reciprocal) were applied, the assumption of normality was not completely satisfied. Given that repeated-measures approach ANOVAs are generally robust to violations of normality, the analyses were conducted using the untransformed data (Field, 2009).

Outliers were identified as (absolute) z-scores > 3, and values were replaced to 2 standard deviations from the mean (Field, 2009). The full-body task had a range of 1–3 outliers across conditions for RT, MT, and nPL, while the upper extremity task had a range of 2–3 outliers across conditions for RT, MT, and nPL. Significance levels were set at *p* = 0.05 and effect sizes were reported using partial eta squared for ANOVAs and Cohen’s *d* for pairwise comparisons.

## 3 Results

### 3.1 Target distances for the full-body task

Targets were placed on the monitor at a distance which corresponded to a lean of mean ± standard deviation for the forward target (142.92 ± 29.50 mm), backward target (80.40 ± 22.51 mm), leftward target (110.05 ± 20.89 mm), and rightward target (116.79 ± 26.22 mm) in the standard condition.

### 3.2 Univariate analyses

**FIGURE 2:**
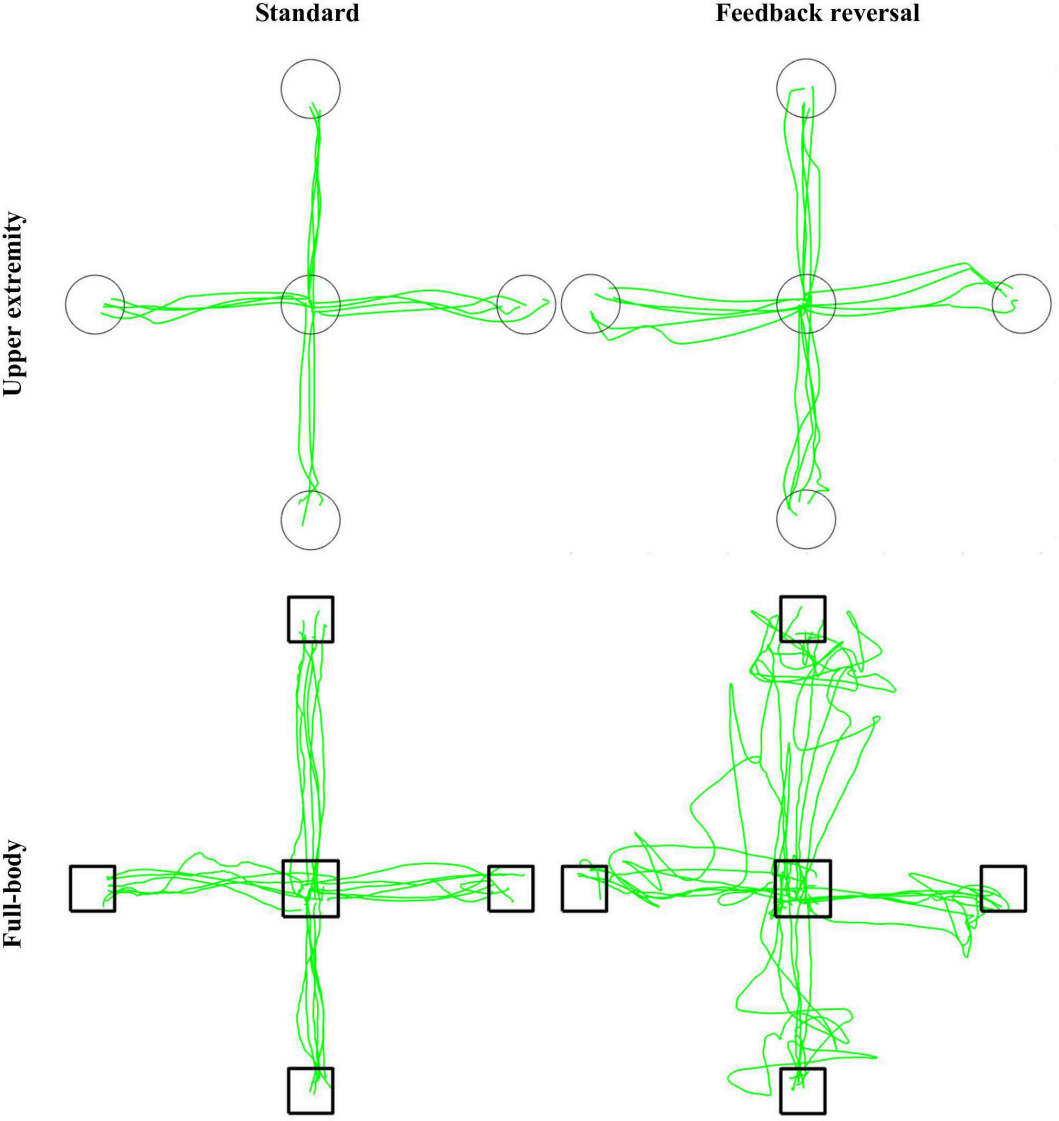
Representative participant for the CMI tasks. Green lines represent cursor trajectories, and circles and boxes represent targets for the upper extremity and full-body task, respectively. Trajectories are shown for all trials for each condition (standard and feedback reversal) within each task (upper extremity and full-body).

**FIGURE 3:**
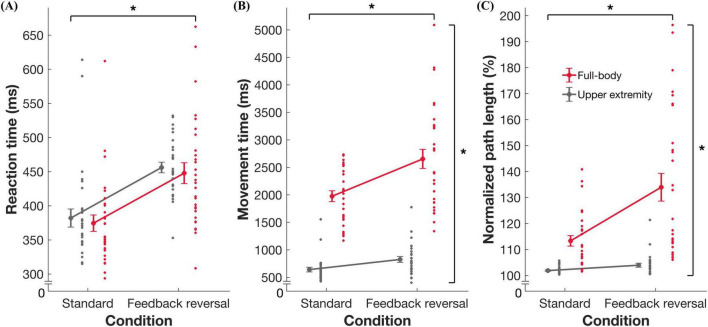
Summary data for task performance. Group mean ± standard error and individual datapoints for **(A)** reaction time, **(B)** movement time, and **(C)** normalized path length for the full-body (red) and upper extremity task (gray). * indicates significance at *p* < 0.05.

ANOVAs revealed an interaction effect of task and condition on MT [*F*_(1_,_28)_ = 9.344, *p* = 0.005, η^2^ = 0.250] and nPL [*F*_(1_,_28)_ = 12.766, *p* = 0.001, η^2^ = 0.313]. *Post hoc* analyses revealed changes across conditions for both the full-body (mean difference [MD] = –658.22, standard error [SE] = 156.82, Cohen’s *d* = –0.78) and upper extremity (MD = –189.63, SE = 28.29, Cohen’s *d* = –1.25) tasks. There were larger increases in MT and nPL across conditions in the full-body task: mean ± standard deviation (MT difference between standard and feedback reversal condition: 658.2 ± 353.1 ms; nPL difference: 20.6 ± 17.7%) when compared to the upper extremity task (MT: 189.6 ± 81.7 ms; nPL: 1.79 ± 1.7%). Furthermore, independent of task, all metrics showed better performance in the standard condition compared to the feedback reversal condition [RT: *F*_(1_,_28)_ = 55.379, *p* < 0.001, η^2^ = 0.664; MT: *F*_(1_,_28)_ = 26.347, *p* < 0.001, η^2^ = 0.485; nPL: *F*_(1_,_28)_ = 17.910, *p* < 0.001, η^2^ = 0.390]. More specifically, the standard condition had faster RTs (373.8 ± 54.2 ms), shorter MTs (1299.5 ± 349.2 ms), and lower nPL (107.5 ± 5.9%) than the feedback reversal condition (RT: 450.8 ± 59.2 ms; MT: 1723.4 ± 566.6 ms; nPL: 118.7 ± 15.6%) (Figures 2, 3 and Table 2).

**TABLE 2:** Mean ± standard deviation for reaction time (RT), movement time (MT), and normalized path length (nPL).

Outcome measure	Full-body		Upper extremity		Task (T)			Condition (C)			T × C		
	Standard	Feedback reversal	Standard	Feedback reversal	*F*_(1,28)_	*p*	η^2^	*F*_(1,28)_	*p*	η^2^	*F*_(1,28)_	*p*	η^2^
RT	370.82 ± 52.8	445.42 ± 76.6	376.77 ± 55.5	456.14 ± 41.8	0.965	0.334	0.033	**55.379**	** < 0.001**	**0.664**	0.091	0.765	0.003
MT	1975.75 ± 532.8	2633.97 ± 885.9	623.16 ± 165.5	812.79 ± 247.2	**217.930**	** < 0.001**	**0.886**	**26.347**	** < 0.001**	**0.485**	**9.344**	**0.005**	**0.250**
nPL	113.14 ± 10.5	133.74 ± 28.2	101.87 ± 1.3	103.66 ± 3.0	**47.352**	** < 0.001**	**0.628**	**17.910**	** < 0.001**	**0.390**	**12.766**	**0.001**	**0.313**

ANOVA results for task and condition effects for RT, MT, and nPL. Bold values indicate significance at the *p* < 0.05 level.

### 3.3 Regression analyses

**FIGURE 4:**
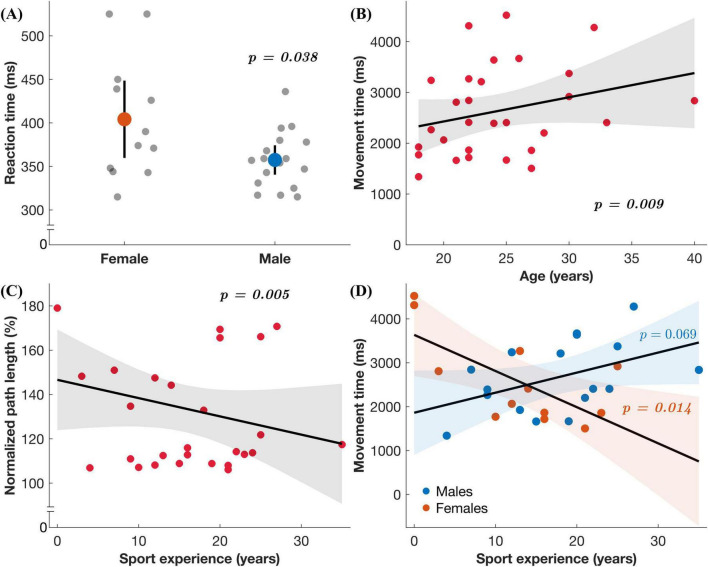
Regression results. **(A)** Reaction time in the upper extremity task under the standard condition as a function of sex. **(B–D)** Each plot includes the regression coefficient (line) and the 95% confidence interval for predicted values (shaded region) in the full-body task under the feedback reversal condition: **(B)** Movement time as a function of age, **(C)** normalized path length as a function of sport experience, and **(D)** movement time as a function of sex (males represented by blue datapoints and females by orange) and sport experience.

Multiple linear regressions found significant associations between exposure variables (age and sport experience), moderating variable (sex) and performance. For the standard condition in the upper extremity task, sex predicted RT (Figure 4 and Table 3), where males had quicker RTs than females (unstandardized *B* = –78.968, *p* = 0.038). For the full-body task, nPL was predicted by sport experience in the feedback reversal condition (Figure 4 and Table 4), whereby more sport experience resulted in less variable nPLs (unstandardized *B* = –3.802, *p* = 0.005). The full-body task also found that age predicted MT in the feedback reversal condition (Figure 4 and Table 5), where older age resulted in longer MTs (unstandardized *B* = 235.546, *p* = 0.009). Lastly, the full-body task also found a sport experience by sex interaction for MT (unstandardized *B* = 203.650, *p* = 0.014) in the feedback reversal condition (Figure 4 and Table 5). A simple slopes analysis revealed that females showed decreases in MT with more sport experience (unstandardized *B* = –91.669, *p* = 0.014), while the relationship between males and sport experience was insignificant. There were no other significant associations.

**TABLE 3:** Regression results for reaction time for the full-body and upper extremity tasks.

Reaction time	Full-body						Upper extremity					
	Standard *R*^2^ = 0.131			Feedback reversal *R*^2^ = 0.289			Standard *R*^2^ = 0.377			Feedback reversal *R*^2^ = 0.439		
	*B*	β	*p*	*B*	β	*p*	*B*	β	*p*	*B*	β	*p*
Age	6.278	0.493	0.446	–10.757	–0.665	0.260	11.124	0.793	0.156	–4.143	–0.505	0.333
Sex^※^	–30.856	–0.238	0.425	1.562	0.009	0.972	–78.968	–0.553	**0.038**	–9.085	–0.109	0.648
Concussion history^Δ^	12.973	0.087	0.797	–75.532	–0.399	0.203	33.019	0.201	0.486	–0.786	–0.008	0.976
Sport experience	–0.732	–0.095	0.825	0.095	0.010	0.980	–1.534	–0.180	0.620	–0.744	–0.150	0.664
Age × sex	–5.021	–0.351	0.718	18.018	0.993	0.266	–15.153	–0.963	0.250	12.580	1.368	0.092
Sport experience × sex	–0.324	–0.031	0.966	–0.437	–0.033	0.960	6.660	0.574	0.352	–2.578	–0.380	0.513
Sport experience × age	0.603	0.541	0.451	–0.062	–0.044	0.946	1.067	0.868	0.161	0.388	0.540	0.351
Age × sex × concussion history	–5.613	–0.318	0.780	–2.010	–0.090	0.931	–1.692	–0.087	0.928	1.043	0.092	0.920
Sport experience × sex × concussion history	4.389	0.324	0.716	–2.094	–0.122	0.880	1.370	0.092	0.903	4.417	0.507	0.481
Sport experience × age × sex × concussion history	–0.672	–0.610	0.507	–0.094	–0.067	0.935	–1.178	–0.970	0.219	–1.053	–1.484	0.055

*N* = 29. In all models, predictor variables were age and sport experience, and moderator variables were sex and concussion history. B = unstandardized beta coefficient; β = standardized beta coefficient. Bold values indicate significance at the *p* < 0.05 level. ^※^ Reference category = females. ^Δ^ Reference category = no history of concussion.

**TABLE 4:** Regression results for normalized path length for the full-body and upper extremity tasks.

Normalized path length	Full-body						Upper extremity					
	Standard *R*^2^ = 0.295			Feedback reversal *R*^2^ = 0.423			Standard *R*^2^ = 0.380			Feedback reversal *R*^2^ = 0.501		
	*B*	β	*p*	*B*	β	*p*	*B*	β	*p*	*B*	β	*p*
Age	1.171	0.544	0.352	4.880	0.865	0.111	0.156	0.556	0.312	–0.040	–0.049	0.920
Sex^※^	0.278	0.013	0.962	3.307	0.058	0.811	0.237	0.083	0.740	1.438	0.172	0.447
Concussion history^Δ^	0.783	0.031	0.919	–0.177	–0.003	0.992	–0.906	–0.276	0.340	–2.498	–0.260	0.318
Sport experience	0.029	0.022	0.954	–3.802	–1.111	**0.005**	0.021	0.123	0.733	–0.018	–0.036	0.911
Age × sex	–1.074	–0.445	0.612	–2.292	–0.362	0.648	–0.214	–0.680	0.411	–1.318	–1.431	0.064
Sport experience × sex	–0.216	–0.122	0.851	4.036	0.865	0.152	–0.024	–0.105	0.863	0.709	1.043	0.068
Sport experience × age	–0.091	–0.483	0.455	–0.141	–0.285	0.625	0.000	–0.020	0.974	–0.026	–0.362	0.504
Age × sex × concussion history	2.115	0.709	0.492	–1.641	–0.210	0.821	–0.291	–0.748	0.440	1.139	1.000	0.255
Sport experience × sex × concussion history	–2.747	–1.201	0.146	–0.530	–0.088	0.903	0.143	0.479	0.527	–0.852	–0.976	0.160
Sport experience × age × sex × concussion history	0.140	0.752	0.366	0.059	0.122	0.870	0.024	0.970	0.218	0.080	1.125	0.117

*N* = 29. In all models, predictor variables were age and sport experience, and moderator variables were sex and concussion history. B = unstandardized beta coefficient; β = standardized beta coefficient. Bold values indicate significance at the *p* < 0.05 level. ^※^ Reference category = females. ^Δ^ Reference category = no history of concussion.

**TABLE 5:** Regression results for movement time for the full-body and upper extremity tasks.

Movement time	Full-body						Upper extremity					
	Standard *R*^2^ = 0.172			Feedback reversal *R*^2^ = 0.252			Standard *R*^2^ = 0.380			Feedback reversal *R*^2^ = 0.166		
	*B*	β	*p*	*B*	β	*p*	*B*	β	*p*	*B*	β	*p*
Age	40.064	0.383	0.542	235.546	1.286	**0.009**	36.840	0.816	0.182	–0.094	–0.002	0.998
Sex^※^	87.597	0.082	0.776	98.726	0.053	0.798	–126.579	–0.276	0.323	–78.724	–0.138	0.635
Concussion history^Δ^	43.863	0.036	0.914	57.316	0.027	0.910	33.138	0.063	0.842	101.488	0.155	0.642
Sport experience	–27.623	–0.436	0.304	–144.131	–1.297	** < 0.001**	0.757	0.028	0.945	–0.198	–0.006	0.989
Age × sex	–43.597	–0.372	0.695	–234.824	–1.143	0.104	–35.151	–0.694	0.445	15.911	0.253	0.790
Sport experience × sex	–23.182	–0.268	0.703	203.650	1.344	**0.014**	9.375	0.251	0.707	20.219	0.436	0.538
Sport experience × age	–4.810	–0.525	0.453	–3.515	–0.219	0.660	2.628	0.664	0.321	2.249	0.457	0.514
Age × sex × concussion history	126.052	0.870	0.437	78.381	0.309	0.698	11.065	0.177	0.867	–16.071	–0.207	0.853
Sport experience × sex × concussion history	–88.760	–0.799	0.363	–51.211	–0.263	0.673	–3.127	–0.065	0.937	–22.111	–0.371	0.672
Sport experience × age × sex × concussion history	4.778	0.528	0.555	–0.178	–0.011	0.986	–3.767	–0.964	0.264	–2.250	–0.463	0.606

*N* = 29. In all models, predictor variables were age and sport experience, and moderator variables were sex and concussion history. B = unstandardized beta coefficient; β = standardized beta coefficient. Bold values indicate significance at the *p* < 0.05 level. ^※^ Reference category = females. ^Δ^ Reference category = no history of concussion.

## 4 Discussion

### 4.1 Comparison of the full-body task vs. upper extremity task

This study investigated how performance is altered when the task requires increased CMI. More specifically, it allowed for the comparison of a CMI-focused full-body balance task with a validated upper extremity task. The slower and more variable performance in the full-body task compared to the upper extremity task could be attributed to the nature of the task itself. Previous work has demonstrated that movement trajectories are more linear when the task uses devices that restrict movement (Okuuchi et al., 2023). For example, single-plane movements when working with touchscreens are more linear than movements performed in three-dimensional space. Therefore, the increased MT and nPL in the full-body task may result from a more curvilinear trajectory due to the complex spatial features in three-dimensions (Figures 2, 3).

The full-body task requires multilimbed coordination of both the upper and lower body, while the upper extremity task was isolated to one arm. This increased number of joints in the full-body task involves more degrees of freedom and increased complexity. MT is likely affected due to the increased coordination demand needed to perform these full-body movements. In addition, the higher degrees of freedom require more inertia, which leads to slower movements. Therefore, when corrective movements were needed to reach the target, the time delay to produce the necessary amount of torque to move the whole body was magnified, evident by the larger differences in MT across conditions within the full-body task (Table 2). This is further supported by work assessing visually-guided movements, which has identified that MT is influenced by the arm component used due to factors such as moment of inertia and joint torque (Hoffmann and Hui, 2010). Movements with distal parts of the arm (compared to proximal components) were performed quicker due to the lower mass and lower inertia. These movements allow for finer motor control and more accurate adjustments based on visual feedback. It is therefore expected that extending beyond the upper arm to include full-body movements, as used in the current study, would lead to increased MTs and more variable movements (increased nPLs). Lastly, the changes evident in the full-body task could also be attributed to the signal-dependent noise theory of motor control (Harris and Wolpert, 1998). This theory suggests that the variability (noise) in a movement increases proportionally with the strength of the motor signal. Larger motor commands, such as moving the full body, would therefore result in increased variability and less precise movements.

### 4.2 Difference between typical dual-task paradigms and CMI paradigms

Previous work has most commonly assessed cognitive influences on balance by implementing a dual-task. A dual-task paradigm consists of assessing an individual’s balance while they are concurrently required to perform a cognitive task, such as standing on a force plate while counting backward by different intervals (Saberi et al., 2024). However, one pitfall of this approach is that it introduces the possibility of task prioritization effects, which occur when an individual consciously or unconsciously prioritizes either the balance task or cognitive task. In the current study, the feedback reversal condition similarly introduces a cognitive component; however, it does not allow the individual the ability to prioritize the motor or cognitive task. Instead, it requires the individual to integrate rules into all aspects of movement as it is impossible to disentangle the two. Therefore, this novel use of an integrated cognitive motor task in a balance setting is beneficial as it could help address some of the pitfalls evident in standard dual-task paradigms.

### 4.3 Factors that influence task performance

The multivariable regressions assessed whether age and sport experience are associated with performance, and whether the relationship is moderated by sex and concussion history. In the full-body task only, younger age and more sport experience predicted shorter MTs and shorter nPLs in the feedback reversal condition, respectively (Tables 4, 5 and Figure 4). Previous research has similarly shown that age and sport experience account for visuomotor performance, more so than concussion history (Dalecki et al., 2019). It is believed that motor developmental stage and skill experience may provide brain network resilience that can compensate for concussion-related performance declines. In addition, the relationship between sport experience and MT in the full-body task was moderated by sex (Table 5 and Figure 4). The results of the study indicated that females saw significant improvements in MT with increasing sport experience; however, this finding did not hold for males. These results suggest that females’ motor performance may benefit more from sport training, while males’ performance is less sensitive to sport experience. These sex differences may be the result of sex-specific motor learning strategies and highlight the need to assess CMI performance as a function of sex. Overall, the alignment of this study with previous findings suggests that age, sport experience, and sex are crucial factors when assessing movements that extend to the full body. However, the lack of association between age and sport experience with the upper extremity task suggests that full-body tasks may provide a more comprehensive assessment. Therefore, the full-body task is more sensitive to factors that influence motor performance and may be more representative of real-world demands.

### 4.4 Future directions

The current study did not find the hypothesized increases in RT between the upper extremity and full-body tasks. Previous work has shown slower RTs in the lower body compared to the upper body (Montés-Micó et al., 2000), suggesting that extending our upper extremity task to the full-body task would replicate this finding. While the methodological approach utilized may be driving these null findings, future work will aim to identify whether the population of interest is a potential factor. While the current study assessed healthy young adults, a critical next step will be investigating whether RT changes become apparent in clinical populations, such as older adults or individuals following concussion.

Future work could use this full-body tool to assess gross motor control and balance, which is affected by a variety of factors such as concussion (Mochizuki et al., 2022), vestibular disorders (Horlings et al., 2009; Cleworth et al., 2020), and aging (Wang et al., 2025). Furthermore, if this task will be implemented in a rehabilitation setting, it is imperative that the training is specifically aimed at the clinical aspects that are intended to be improved. Previous work focusing on the functional recovery of activities of daily living following stroke found that a leg-focused rehabilitation group had larger improvements in their recovery compared to an arm-focused group (Kwakkel et al., 1999). Therefore, if the goal is to assess gross motor control and its influence on activities of daily living, then a more ecologically valid full-body task may lead to an improved ability to detect differences.

### 4.5 Strengths and weaknesses

Among the key strengths of this study is the expansion of the upper extremity task to a full-body CMI task while remaining ecologically valid. Unlike the isolated upper extremity task, which primarily relies on visual and proprioceptive input, a full-body approach relies additionally on continuous information from vestibular input. While focusing on coordination between the upper and lower body, rather than isolated limb control, this full-body assessment enhances our understanding of CMI in a realistic and functionally relevant manner. In addition, the use of a within-subjects design controlled for individual variability and allowed for a direct comparison to the previously validated upper extremity version. Lastly, this novel cognitive-integration approach in a balance task may compensate for the traditional drawbacks that emerge in a dual-task balance assessment. Building upon the valuable insights provided by the study, several limitations should also be acknowledged. Firstly, the sample of healthy young adults limits the generalizability of the results to other groups, such as older adults or clinical populations. Secondly, the low number of individuals with concussion history may not have provided enough statistical power to identify previous concussions as a significant predictor in the regression model (Tables 3–5). Lastly, the full-body task involves multiple sensory inputs, leading to difficulties in pinpointing the exact source of any deficits. However, this complexity allows for the ability to model real-world tasks, where multi-sensory integration is both inherent and integral in these tasks.

## 5 Conclusion

The addition of a full-body CMI task provides a more comprehensive analysis of sensory, motor, and cognitive contributions to coordination tasks. This study provides additional insight into how balance is affected and controlled when CMI is challenged. Overall, the upper extremity task may be limited in its ability to extract meaningful information that might contribute to a clinical population’s difficulty in performing activities of daily living. Therefore, the use of this task in clinical populations has the potential to uncover differences that might not be apparent in the standard assessment protocol.

## Data Availability

The raw data supporting the conclusions of this article will be made available by the authors, without undue reservation.
